# Protein–Ligand Binding and Structural Modelling Studies of Pheromone-Binding Protein-like Sol g 2.1 from *Solenopsis geminata* Fire Ant Venom

**DOI:** 10.3390/molecules29051033

**Published:** 2024-02-27

**Authors:** Siriporn Nonkhwao, Erika Plettner, Sakda Daduang

**Affiliations:** 1 Faculty of Pharmaceutical Sciences, Khon Kaen University, Khon Kaen 40002, Thailand; siriphorn_nonkhaow@kkumail.com; 2 Department of Chemistry, Simon Fraser University, Burnaby, BC V5A 1S6, Canada; 3 Protein and Proteomics Research Center for Commercial and Industrial Purposes (ProCCI), Khon Kaen University, Khon Kaen 40002, Thailand

**Keywords:** *Solenopsis geminata*, venom protein Sol g 2.1, pheromone-binding proteins (PBPs), protein–ligand binding

## Abstract

Sol g 2 is the major protein in *Solenopsis geminata* fire ant venom. It shares the highest sequence identity with Sol i 2 (*S. invicta*) and shares high structural homology with LmaPBP (pheromone-binding protein (PBP) from the cockroach *Leucophaea maderae*). We examined the specific Sol g 2 protein ligands from fire ant venom. The results revealed that the protein naturally formed complexes with hydrocarbons, including decane, undecane, dodecane, and tridecane, in aqueous venom solutions. Decane showed the highest affinity binding (K_d_) with the recombinant Sol g 2.1 protein (rSol g 2.1). Surprisingly, the mixture of alkanes exhibited a higher binding affinity with the rSol g 2.1 protein compared to a single one, which is related to molecular docking simulations, revealing allosteric binding sites in the Sol g 2.1 protein model. In the trail-following bioassay, we observed that a mixture of the protein sol g 2.1 and hydrocarbons elicited *S. geminata* worker ants to follow trails for a longer time and distance compared to a mixture containing only hydrocarbons. This suggests that Sol g 2.1 protein may delay the evaporation of the hydrocarbons. Interestingly, the piperidine alkaloids extracted have the highest attraction to the ants. Therefore, the mixture of hydrocarbons and piperidines had a synergistic effect on the trail-following of ants when both were added to the protein.

## 1. Introduction

The tropical fire ant (*Solenopsis geminata*) is one of the ubiquitous ant species in Thailand. They are known as an aggressive ant species due to their behavior and venomous painful sting. The venom is produced in the venom glands, stored in the poison sac, and then secreted through a sting at the tip of the abdomen. The venomous secretion comprises 90–95% basic piperidine alkaloids and four major allergen proteins [1]. The piperidine alkaloids are mainly 2-methyl-6-alkyl piperidines with different lengths of alkyl or alkenyl chains [2]. The alkaloids are used in defense against possible predators. Furthermore, venom alkaloids help these ants control and avoid competition for their hosts [3]. Derivatives of solenopsins, which are the fire ant potent piperidine alkaloids, have been found to have antibacterial, antifungal, insecticidal, and antiangiogenics activities [4,5,6,7]. As mentioned, the *S. geminata* venom also contains four major proteins, including Sol g 1, 2, 3, and 4, which are responsible for allergenic activity [8]. Importantly, Sol g 2 is one of the major protein components in the venom [9].

Sol g 2.1 protein (GenBank: UYX46120.1) shares an 83.05% sequence identity with Sol i 2 (*S. invicta*); both consist of five α-helices and three intramolecular disulfide bridges, forming a hydrophobic cavity. Interestingly, Sol i 2 has a high binding affinity with hydrophobic molecules such as (*E*)-β-farnesene aphid alarm pheromone, plant volatiles, analogs of ant trail pheromones like decane and undecane, and short fatty acids [10]. Moreover, the three-dimensional structure of Sol i 2 is highly similar to odorant-binding proteins (OBPs), which are located in the olfactory organs of various insect species [11]. OBPs are small soluble proteins consisting of 130–150 amino acids, and they are found in the sensillum lymph of insects [12,13]. These proteins are mainly involved in the peripheral olfactory system, in the sensillum lymph fluid, by acting as a mediator between odorants and their membrane receptors [14,15]. The OBP family also includes proteins that specifically bind semiochemicals, e.g., pheromone-binding proteins (PBPs), which transport and scavenge pheromones to activate and protect pheromone receptors (PRs). PBPs are acidic proteins that contain 120–150 amino acids and six conserved cysteine residues. Interestingly, Sol g 2.1 and LmaPBP (PBP from the cockroach, *Leucophaea maderae*) share high structural homology and an inner hydrophobic cavity [10]. Thus, Sol g 2.1 protein may be involved in binding and transporting hydrophobic molecules like ant pheromones or straight-chain alkyl substituents of piperidine alkaloids to solubilize them in aqueous environments [10,11,13,16]. Nevertheless, specific Sol g 2.1 protein ligands in *S. geminata* crude venom have not been reported. In this research, we isolated endogenous ligands of Sol g 2.1 protein in the fire ant crude venom by gel filtration and then investigated the binding of these endogenous compounds to Sol g 2 and their function further. Using rSol g 2.1, we studied its binding of endogenous ligands from venom using a competitive binding assay. In addition, we predicted the structure of the protein complexed with various ligands by molecular docking simulations. The function of Sol g 2.1 protein and its endogenous ligands were examined by a trail-following bioassay.

## 2. Results

### 2.1. Piperidine Alkaloid Profiles from S. geminata Venom Extraction

**Figure 1:**
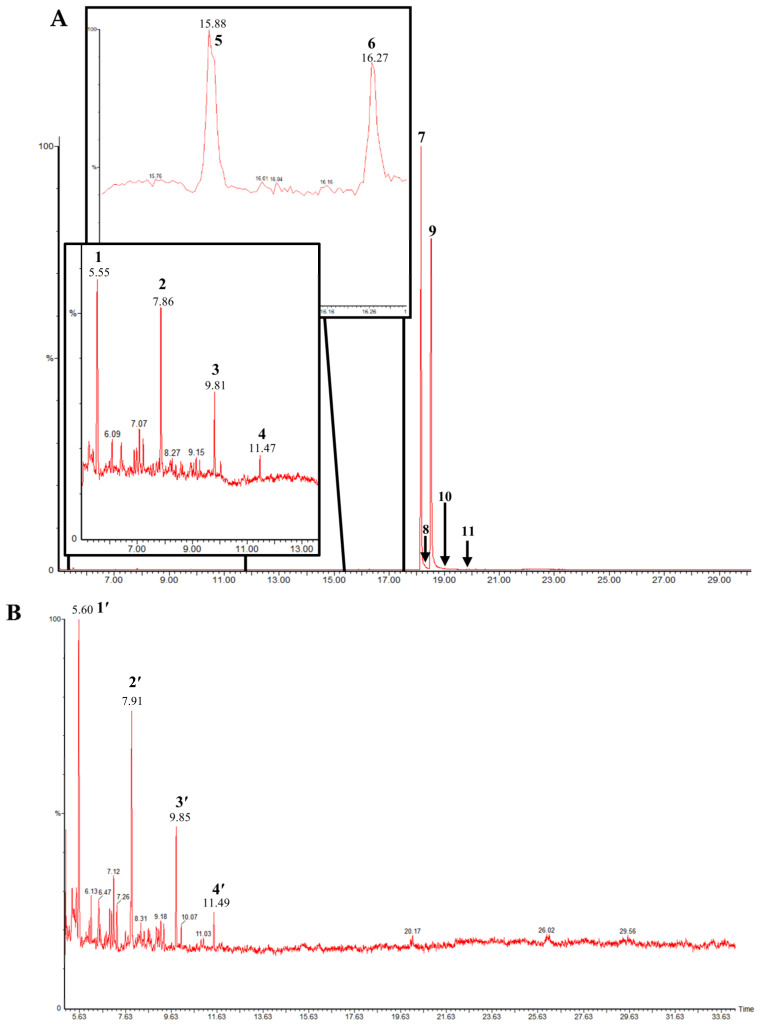
(**A**) GC/MS chromatogram of the *S. geminata* crude venom extraction. (**B**) GC/MS chromatogram of each compound after being separated by P-2 gel column.

The venom contained approximately 0.08 µg/µL of the total protein concentration. After extraction, the organic solution was then analyzed on GC/MS. The results revealed that there were 11 peaks detected (Figure 1A). Peaks 1 to 4 were hydrocarbons, including decane, undecane, dodecane, and tridecane, which were identified using straight-chain hydrocarbon standards by comparing the retention times and mass spectra. Piperidine alkaloids were detected at peaks 5–11. Peaks 5 and 6 had mass spectra corresponding to *cis-* and *trans*-C9 (2-methyl-6-n-nonylpiperidines), respectively. Peaks 7 and 9 had base and molecular mass ions at *m*/*z* 98 and 252 [M+], respectively, corresponding to *cis*- and *trans*-2-methyl-6-n-undecylpiperidines, also known as solenopsin A and isosolenopsin A, both prominent compounds in fire ant venom (90% and 9.2%, respectively, Table 1). Peak 8 had ions *m*/*z* 96 and 111, consistent with 1,6-didehydro-2-methyl-6-undecylpiperidine. The mass spectrum also showed a base peak ion at 98 *m*/*z* and a mass at 252 *m*/*z* [17,18]. Moreover, the mass spectra of peaks 10 and 11 showed a base peak at ion 98 *m*/*z* and mass at 281 *m*/*z*. After comparing with previous reports, we found that these peaks could correspond to *cis-* and *trans*-C13 (2-methyl-6-n-tridecylpiperidines), respectively (Figure S1 and Table 1) [19].

### 2.2. Binding Assay

**Figure 2:**
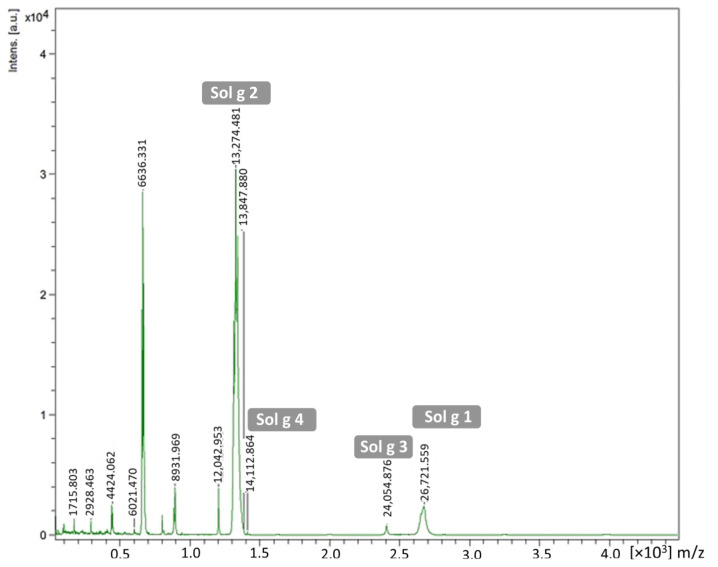
MALDI-TOF MS spectrum of the through-flow protein from the crude venom after separation on gel-filtration column.

After the complexes of proteins and ligands and any free ligands in the crude venom were separated using a gel-filtration column, the flow-through solution was then extracted and analyzed on GC/MS. The chromatogram showed that there were four peaks, including 1′, 2′, 3′, and 4′ detected (Figure 1B). These compounds were decane, undecane, dodecane, and tridecane, with peak area ratios of 45%, 36%, 15%, and 4%, respectively. To identify the protein content in the flow-through solution, MALDI-TOF MS was performed on this procedure. We found that the proteins forming the fire ant crude venom were major at 13,274.48, followed by 14,112.86 and 24,054.88 Da, parallel to Sol g 2, Sol g 4, and Sol g 3, respectively. Sol g 1 protein fragments were shown at peaks 26,721.56 and 6636.33 Da [9,20]. The results showed that Sol g 2 is a major protein in *S. geminata* venom (Figure 2). Furthermore, the through-flow solution was separated using SDS-PAGE. From the results, we found that at approximately 15 kDa (band C), 37 kDa (band A), and 26 kDa (band B), these corresponded to Sol g 2, Sol g 1, and Sol g 3, respectively (Figure S2) [9,21]. The expected band of Sol g 2 protein (band C) was found to be identical to the venom protein Sol g II (Accession AAY32926.1), which is an allergen protein in *S. geminata* venom (Table 2).

### 2.3. Fluorescence Binding Assay

**Figure 3:**
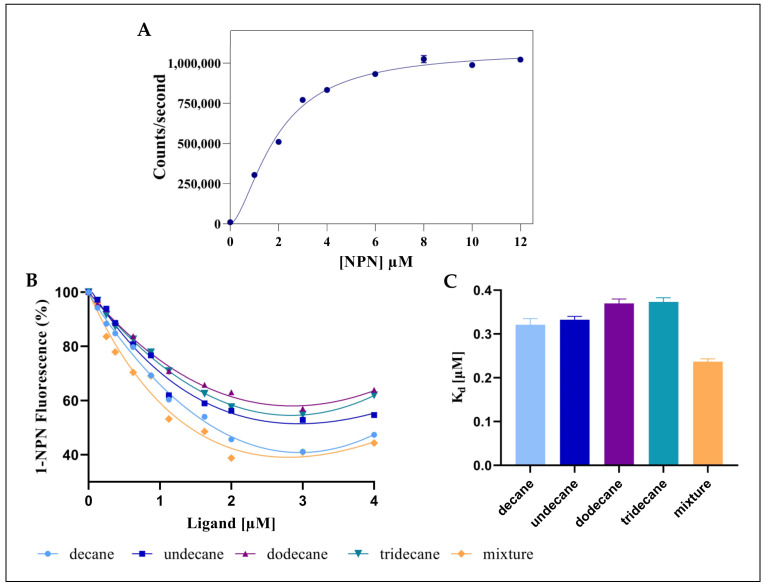
(**A**) Plot of fluorescence intensity (Counts/second) vs. concentration of NPN added to a sample of Sol g 2.1 protein. The points represent the average fluorescence intensity at the maximal emission wavelength (400 nm) ± SEM, triplicates. The curve was fitted using a nonlinear single-binding fitting model since the latter gave a better fit (R^2^ = 0.98). (**B**) Competitive binding curves of selected ligands. (**C**) K_d_ values of competitor ligands with Sol g 2.1 protein.

The fluorescent emission spectra revealed a maximum emission peak at 337 nm for all conditions [22,23]. However, when the cleaned recombinant Sol g 2.1 was combined with different doses of NPN, a significant emission peak at 400 nm was seen. The fluorescence spectra at the maximum signal intensities of 400 nm were obtained from the titration of various concentrations of NPN, ranging from 0 to 12 µM. As the NPN concentration increased, the isotherm reached saturation, and the data were then fitted to a specific binding with the Hill slope model. The K_d_ and h slope of the rSol g 2.1 protein and NPN were 1.90 ± 0.08 µM and 1.64 ± 0.12, respectively (Figure 3A). In our finding, the affinity value of the Sol g 2.1 protein with NPN was within the range seen with other insect OBPs [24]. The reduction in fluorescence intensity at 400 nm was evaluated to assess the binding affinities of Sol g 2.1 protein with the competitive ligands. The results of decane, undecane, dodecane, and tridecane, as the NPN displacing ligands, were shown as percentages of the NPN fluorescence reduction (Figure 3B). The K_d_ values of decane, undecane, dodecane, and tridecane of the rSol g 2.1 protein binding were 0.32, 0.33, 0.39, and 0.38 µM, respectively (Figure 3C). According to the findings, decane had the highest affinity for interacting with the rSol g 2.1 protein, followed by undecane, dodecane, and tridecane. This is consistent with the gel-filtering results, which showed that the hydrocarbons eluted were 45% decane, 36% undecane, 15% dodecane, and 4% tridecane. Interestingly, the K_d_ value of the mixture of hydrocarbons binding to rSol g 2.1 protein was reduced to 0.24 µM. These findings imply that the protein has a stronger affinity for the combination than the individual ligands, indicating a positive blend effect. This result is relative to the equilibrium constant fitting with the Hill slope, which has a h value higher than 1.0, meaning that there is more than one binding site with positive cooperativity between the protein and ligands [25].

### 2.4. Sol g 2.1 Protein Homology Modeling and Molecular Docking

**Figure 4:**
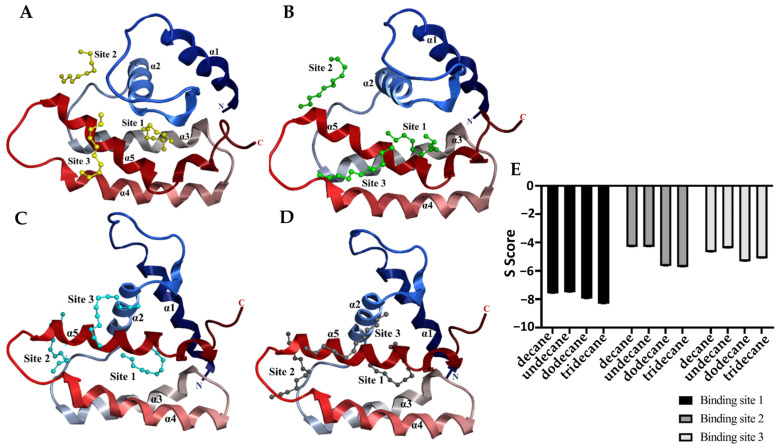
Molecular docking of the endogenous ligands binding at different Sol g 2.1 model binding sites. (**A**–**D**) Show the top ranks of binding sites, including internal (site 1) and external (site 2–3) binding sites in the Sol g 2.1 protein model, which were contacted with the ligands. Decane, undecane, dodecane, and tridecane ligands were represented as yellow, green, cyan, and dark gray colored sticks, respectively. (**E**) The top rank of the lowest S score of each ligand at the three binding sites, triplicate (S score mean ± SEM).

**Figure 5:**
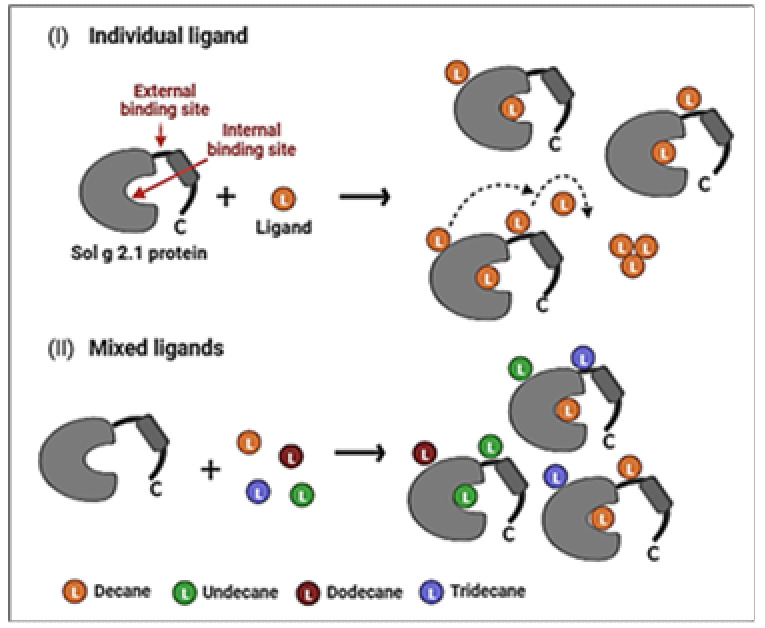
An illustration diagram of the possible roles of a positive blend effect of ligand binding at Sol g 2.1 protein binding sites. (**I**): a role of individual ligand and Sol g 2.1 protein binding. The ligands prefer binding to the inner hydrophobic cavity to the external binding site. Ligands situated in external binding sites have the potential to shift and engage in self-binding form (dash arrows). (**II**): shows a role of the positive blend effect of Sol g 2.1 protein with hydrocarbon ligands. The ligands can bind at both sites of the Sol g 2.1 protein model (Created with BioRender.com).

The model generated for the Sol g 2.1 protein from the SWISS-MODEL, which exhibits the highest structural similarity (82.35%) with the Sol i 2 (template, 2ygu.1.A), was utilized for the molecular docking of protein and ligand binding (Figure S3). To predict the binding sites of the endogenous ligands (decane, undecane, dodecane, and tridecane) in Sol g 2.1 protein, we used molecular docking (Figure 4). At the internal binding site 1 (PLB = 2.38), all ligands of alkanes were surrounded by mostly non-polar amino acids, including Trp36, Met40, Val61, Ile65, Ile79, Ile104, Val109, and Val110 of Sol g 2.1. For the longer hydrocarbon chains, more non-polar amino acid residues were in contact with these ligands. Val45 interacted with undecane, dodecane, and tridecane, as well as Ile66, and contacted with decane and tridecane. Moreover, Leu105 also surrounded the dodecane ligand. However, the Tyr46, Asn58, Cys62, Cys75, Thr101, and Thr113 amino acid residues of the Sol g 2.1 protein pocket also interacted with all ligands (Figure S4). The average S scores of decane, undecane, dodecane, and tridecane binding to Sol g 2.1 were −7.58 ± 0.02, −7.51 ± 0.01, −7.96 ± 0.02, and −8.30 ± 0.03, respectively (triplicates, mean ± SEM). At the external binding site 2 (PLB = 0.61), decane and undecane were in contact with mostly polar amino acids, including His37, Tyr46, Asp47, Ans93, and Arg94, as well as non-polar amino acid residues, which were Ala41 and Pro49 (undecane). This binding site was located around the α1-α2 and α4 regions. Next, dodecane and tridecane interacted with both polar and non-polar amino acids, which were lined between the loop among the α2-α4 regions. Tyr46, Asp47, Asn48, Thr87, Asn93, Arg94, and Lys96 were polar residues at this binding site. There were also some non-polar residues consisting of Pro49, Ile54, Ala97, and Ile100 (dodecane). At this binding site on the Sol g 2.1 protein model, the average S scores of the decane, undecane, dodecane, and tridecane ligands with the protein complexes were −4.31 ± 0.01, −4.30 ± 0.01, −5.66 ± 0.01, and −5.72 ± 0.01, respectively (triplicates, mean ± SEM). At binding site 3 (PLB = 0.03), all ligands were in contact with the polar amino acids that included Arg85, Glu86, Asn99, Gly102, Cys103, and Arg106, and additionally, Arg89 for undecane and tridecane. There was also Val33 for the non-polar residue interaction of all ligands. Moreover, Ile98 and Leu105 bound with decane and two ligands, including dodecane and tridecane, respectively. This binding site was located near the C-terminus and helices α3-α4. The S scores of all ligands were −4.68 ± 0.02, −4.41 ± 0.01, −5.33 ± 0.01, and −5.12 ± 0.01, respectively (Figure 4E). With the prediction of Sol g 2.1 and ligand complexes, we found that the lowest S score of the protein and each ligand was at the internal binding site (site 1), suggesting that these ligands are more stabilized in the inner hydrophobic pocket of the protein by a hydrophobic interaction than at other sites (Figure 5).

### 2.5. Trail-Following Bioassay

**Figure 6:**
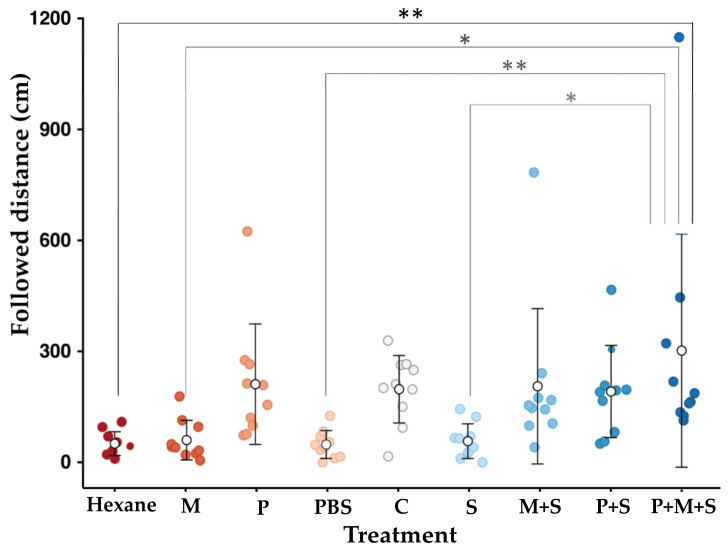
Response of *S. geminata* workers (*n* = 10) exposed to each treatment in 10 min. Colored and white dots show the distance that each ant and 10 ants on average (mean ± SEM) traveled following trails, respectively. Hexane = only hexane; M = mixture of medium-chain hydrocarbons (C10-13); P = piperidine alkaloid extracted from *S. geminata* venom; PBS = PBS buffer pH 7.4; C = *S. geminata* crude venom; S = rSol g 2.1 protein; M+S = mixture of medium-chain hydrocarbons in rSol g 2.1 protein; P+S = piperidine alkaloids extracted in rSol g 2.1 protein; P+M+S = piperidine alkaloids extracted mixed with medium-chain hydrocarbons and rSol g 2.1 protein. Asterisk (*) means statistically different (Tukey’s honest significant difference (HSD) test, * p < 0.05 and ** p < 0.01).

The average distances (cm ± SEM) of the trail-following by the ant workers to M, P, C, M+S, P+S, and P+M+S were 60.0 ± 16.9, 221.1 ± 51.5, 197.5 ± 28.8, 205.6 ± 66.4, 191.1 ± 39.4, and 303.4 ± 99.6, respectively. The fully reconstituted venom (P+M+S) is the most attractive for the worker *S. geminata* ants when following only piperidine alkaloids (P), the reconstituted treatment (M+S), crude venom (C), and piperidine in the rSol g 2.1 solution (P+S). Moreover, the ants followed the fully reconstituted treatment for longer distances than the negative control groups, which did not elicit any response from the ants (Figure 6).

## 3. Materials and Methods

### 3.1. Harvesting and Extraction of S. geminata Crude Venom

To obtain *S. geminata* venom for the studies, adult workers were harvested from Khon Kaen City in Thailand. After stimulation, their venom was collected drop-by-drop using a capillary tube, and the crude venom was then dissolved in PBS buffer, pH 7.4 (1.8 mM KH_2_PO_4_, 2.7 mM KCl, 137 mM NaCl, and 10 mM Na_2_HPO_4_). Next, the crude venom was extracted in double volume of 1% ethyl acetate in distilled hexane. The mixture was then shaken until it was completely separated into two layers. An organic phase was transferred into a new test tube with Na_2_SO_4_ for drying. After that, the extracted solution was analyzed by gas chromatography coupled with mass spectrometry detection (GC/MS) on a Clarus 690-GC interfaced with a Clarus SQ8T-MS (Perkin Elmer, Waltham, MA, USA). The GC was equipped with a 30 m fused silica SBP-5 column (0.25 mm i.d., 25 µm film thickness, Supelco, Bellefonte, PA, USA), and He was used as the carrier gas (1.0 mL/min). The GC was programmed as follows: 80 °C for 5 min at the initial temperature, 10 °C/min to 250 °C (hold for 15 min). The split/splitless injector was set to 250 °C and operated in splitless mode. The ionization was accomplished using electron impact (EI), and the MS scan was collected between *m*/*z* = 50–450.

### 3.2. Binding Assay

The binding of endogenous ligands to Sol g 2.1 in the crude venom was investigated by size-exclusion chromatography (P-2 gel, BioRad, molecular size limit 2 kDa) for separating large molecules (protein–ligands complexes) from smaller molecules (free ligands) [26]. In this experiment, 50 mg of gel beads was added into a small pipette tip (200 µL capacity) fitted with a cotton plug. PBS buffer (50 µL) was then added to swell the gel. We applied 50 µL of the *S. geminata* crude venom onto the column, and the elution fraction from a column bed was then collected. After the column was washed with 50 µL of PBS buffer, the flow-through solution was pooled into the existing filtrate. The combined flow-through solution was carried out for the protein identification and analysis of endogenous ligands and Sol g 2 protein on GC/MS and SDS-PAGE, and MALDI-TOF MS and LC-MS/MS, respectively. Specifically, the flow-through was extracted and analyzed on GC/MS, as described above.

To identify the Sol g 2 protein, the flow-through solution was analyzed on one-dimensional SDS-PAGE. The molecular mass of the proteins was then determined using the matrix-assisted laser desorption/ionization coupled to time-of-flight mass spectrometry (MALDI-TOF MS) (Bruker Launches autoflex (TM) speed MALDI-TOF(/TOF)). The autoflex TOF/TOF (Bruker Daltonics flexAnalysis) was used to examine the mass spectra. The linear operation mode was used to determine the acquisition settings, and the positive polarity and total 3000 spectra were summed. Afterward, the expected band of the Sol g 2 protein on the SDS-PAGE was cut, extracted, and proteolyzed by trypsin, followed by liquid chromatography and tandem mass spectrometry by LC-MS/MS [9]. Peptides were separated on a nano-liquid chromatography system (EASY-nLC II, Bruker, Madison, WI, USA). The sample was loaded onto an EASY-Colum (10 cm, i.d., 75 μm, 3 μm, C18-A2, Thermo Scientific, Boston, MA, USA) using 0.1% formic acid in water and acetonitrile for mobile phases A and B, respectively. The LC was coupled to a nano-spray ESI-Ion trap MS (Sciex tripletof^®^ 6600+) and a time-of-flight (TOF) analyzer for the MS/MS scan mode. The PEAKS DB Protein Identification LC-MS/MS Software (PEAKS Studio 10.6) was used to identify peptides from the peaks [27].

### 3.3. Expression of Sol g 2.1 Protein in E. coli

The Sol g 2.1 coding sequence (GenBank: UYX46120.1) in the pProEx-HTB expression vector, which is composed of His6-tagged protein at the C-terminus, was expressed in *E. coli* BL21 (DE3) pLysS competent cells (Promega, Selangor, Malaysia), as described previously [22]. A single colony was inoculated in Luria–Bertani (LB) medium containing 50 μg/mL of Ampicillin at 37 °C overnight. The cell culture was induced with isopropyl β-D-1-thiogalactopyranoside (IPTG). After harvesting the cells, the cell pellets were extracted in a lysis buffer (80 mM Tris-HCl, 200 mM NaCl, 1 mM EDTA, and 4% glycerol, pH 7.2). The protein accumulated largely in insoluble inclusion bodies; it was then refolded using 8 N guanidinium HCl [23]. After denaturation and renaturation, the soluble protein solution was purified using a nickel affinity column (His-Bind resin, Novagen, Madison, WI, USA) using 20 mM Tris-HCl pH 7.4, 500 mM NaCl, and 20 mM imidazole as a binding buffer. His6-tagged proteins were eluted by increased concentrations of imidazole (50–500 mM) following the desalting step. For delipidation, the purified protein was incubated with methyl-functionalized methacrylate HIC resin (hydrophobic interaction chromatography, Bio-Rad, Hercules, CA, USA) in 50 mM Tris-HCl pH 7.4 at 4 °C for three days on a rotary mixer [28].

### 3.4. Fluorescence Competitive Binding Assay

The fluorescence binding assay of rSol g 2.1 protein was conducted using the fluorescent probe N-phenyl-1-naphtylamine (NPN). To investigate the affinity binding, a stock of 1 mM NPN in methanol was titrated into 2 µM rSol g 2.1 in 50 mM Tris-HCl (pH 7.4) to a final concentration range of 0–12 µM by using methanol as a negative control. The fluorescence intensity was measured on a PTI QuantaMaster fluorometer (Horiba Ltd., Kyoto, Japan) with a 337 nm excitation wavelength and emission scan ranging from 300–500 nm at room temperature in triplicate. To obtain the equilibrium dissociation (K_d_) value, the fluorescence intensity at the maximal emission wavelength 400 nm was plotted for each NPN concentration. Data were fitted to a specific allosteric binding model using GraphPad Prism Version 9.0 (GraphPad Software, San Diego, CA, USA). Y = B_max_ × X^h^/(K_d_^h^ + X^h^) was used as an equation in the fitting model, where B_max_ is the maximal specific binding in the same unit as Y, X is the NPN concentration (µM), and Y is the fluorescence intensity counts/second. Moreover, h is the Hill slope, which is 1.0 for cases with one site. If there is more than one binding site per protein and there is cooperativity, then h > 1.0, and the graph takes on a sigmoidal appearance.

The binding of the endogenous ligands, including decane, undecane dodecane, and tridecane, to rSol g 2.1 protein was measured by a competitive binding assay. In this procedure, 1 mL of rSol g 2.1 protein (2 µM) in 50 mM Tris-HCl (pH 7.4) containing NPN (4 µM) was titrated with each ligand (1 mM in methanol stock) to the final concentrations of 0, 0.125, 0.375, 0.625, 0.875, 1.125, 2, 3, and 4 µM. Additionally, the same concentrations of the blended ligands, which consisted of decane (45%), undecane (36%), dodecane (15%), and tridecane (4%), were used to investigate the blend effect of Sol g 2.1 protein. The resulting isotherms were fitted (GraphPad Prism 9) to both a one-site model and an allosteric model. Where K_d_ is the equilibrium dissociation constant (µM), the ternary complex constant is alpha; when alpha = 1.0, the modulator does not affect binding, and when alpha is less than 1.0, the modulator decreases ligand binding.

### 3.5. In Silico Studies, Homology Modeling, and Molecular Docking of Sol g 2.1 Protein and Ligands

The homology model of Sol g 2.1 protein, which is based on the crystallized Sol i 2 (*S. invicta*, PDB ID: 2ygu.1.A, 2.60 Å resolution) template was generated by using the SWISS-MODEL program (https://swissmodel.expasy.org/, accessed on 20 January 2022) [29]. Initially, the Sol g 2.1 protein structure was set up by inputting the acids sequence and running it via the SWISS-MODEL template library (SMTL) for template searching. Next, the top-ranked template, which has the highest sequence and structural similarity, was selected and built the model [28]. The docking of Sol g 2.1 and the endogenous ligands were simulated using MOE version 2019 (Molecular Operating Environment). MOE protonate 3D, the three-dimensional structural (3D) model of Sol g 2.1 protein, was protonated by the ionization state and adding hydrogen atoms to the structure, as described previously [23]. Afterward, in the energy minimization step, the protein was energy-minimized with the rigid water molecule constraints in the Amber 10 force field. Next, the ligand site finding was based on the Alpha Shapes center approach using the MOE Site Finder [30]. Each ligand was placed at the top of positive PLB ranks via dummy atoms. Each site was placed as per the Triangle Matcher method, and the complex was scored at 30 poses using the London dG score tool. The induced fit energy minimization model was refined at 5 poses using the GBVI/WSA force field in which the ligand’s free energy binding was computed in the S score [31]. The structure with the lowest S score is the best pose of the binding affinity model [32]. The top rank of the MOE docking S scores with the lowest RMSD (root-mean-square deviation of the atomic positions) value of the Sol g 2.1 protein and various ligands were chosen (triplicates, average ± SEM (standard error of the mean)).

### 3.6. Trail-Following Bioassay

The *S. geminata* colony was collected from Mueang Khon Kaen District, Khon Kaen, Thailand. The ants were acclimatized by being placed in a plastic cage box at room temperature in an open-air environment. They were fed with 20% *w*/*v* of sugar in water and frozen crickets [33]. Venom was collected drop-by-drop from 60 individual ants (each ant had an average of 20 drops) and was then dissolved in 60 µL of PBS buffer, as described previously [9]. After harvesting, the protein concentration of crude venom was measured by Bradford’s method. In this procedure, a positive control group was prepared from 1 µL of the crude venom in 25 µL of PBS buffer (treatment C; 1 ant equivalent (AE)). Next, 40 µL of the crude venom stock was aliquoted and extracted in 80 µL of 1% ethyl acetate in hexane, as described above. The upper phase was taken out into a new vial, which was called piperidine alkaloids and organic compounds. Afterward, 2 µL of the extracted solution was dissolved in 25 µL of hexane (treatment P; 1 AE). Moreover, a reconstituted venom was constructed from 2 µL of the piperidine alkaloids extracted in 25 µL of rSol g 2.1 protein in PBS to 1 ng/µL of the final concentration (treatment P+S; 1 AE). A mixture of medium-chain hydrocarbons, including decane (45%), undecane (36%), dodecane (15%), and tridecane (4%), was used as representatives of the endogenous ligands bound to Sol g 2 protein in *S. geminata* venom. Each compound was dissolved in hexane to 1 ng/µL of the final concentration (treatment M; 1 AE). In addition, the mixture of hydrocarbons was aliquoted into rSol g 2.1 protein in PBS buffer to 1 ng/µL of the final concentration, which gave the artificially reconstituted trail pheromone-like with the protein (treatment M+S). A fully reconstituted *S. geminata* venom was composed of 2 µL of piperidine alkaloids extracted in rSol g 2.1 protein in PBS and the mixture of hydrocarbons, which were 1 ng/µL in a final concentration for each compound (treatment P+M+S; 1 AE). There were three negative control groups, including hexane, PBS buffer, and cleaned 1 ng/µL rSol g 2.1 protein in PBS buffer (S).

In the trail-following bioassay, each test stimulus was administered as 1 µL per arc (or 26 drops for the full circle) by using a micro syringe along the perimeter of a circular Whatman filter paper (90 mm in diameter, Sigma-Aldrich, MO, USA). The circular filter paper was marked with a circle 1 cm from the edge, and the circle was divided into 26 arcs. To begin the bioassay, the treated paper was placed in the center of an acrylic arena (30 cm × 17 cm × 10 cm), and a Falcon tube containing a single worker ant was placed 2.5 cm from the edge of the paper. Each ant was given 5 min in the arena to settle down before filming. The ant’s movement was tracked for 10 min (*n* = 10) [33,34,35].

### 3.7. Statistical Analysis

GraphPad Prism 9 (GraphPad Software, San Diego, CA, USA) was used to analyze all data. R studio (version 2022.07.1) was used to visually generalize the ant-following-distance response to all treatments for the bioassay analysis. ANOVA was used to examine the variance and significance, with Tukey’s honest significant difference (HSD) test, * p < 0.05 and ** p < 0.01.

## 4. Discussion

The crude venom from the fire ant *S. geminata* consists of various components, such as piperidine alkaloids, pheromones, fatty acids, small hydrophobic compounds, and proteins, and among them, the Sol g 2 protein, which is the major allergen protein in *Solenopsis* spp. venom. This protein has an inner hydrophobic pocket, which can bind with hydrophobic compounds. Moreover, many previous studies have reported that the three-dimensional structure and physiochemical properties of *Solenopsis* venom allergen proteins, Sol i 2 and Sol g 2.1, are similar to PBPs [9,10,16]. The protein may act as a pheromone transporter protein from the site of pheromone biosynthesis to the sting apparatus and beyond after the sting [10,16]. However, there is no report about specific endogenous ligands of this protein in *S. geminata* venom after secreting. Here, we present the first report to investigate the specific binding activity and ligands of the protein.

The alkaloid peaks are highly dominating in the hexane extracts of fire ants. Because the chemical structures and GC profiles of piperidine and piperideine alkaloids in fire ant venom are well-defined, the chemical identities of major peaks can be determined by comparing the peak characteristics with previously published profiles of alkaloids of the two parental species [36]. From the piperidine alkaloid profiles, we found that solenopsin A and isosolenopsin A were the major components in the venom from *S. geminata*, which is consistent with the results from previous studies [17,18,35]. The binding assays showed that decane was the major endogenous ligand, followed by undecane, dodecane, and tridecane, respectively, which was an unexpected finding. Surprisingly, the piperidine alkaloids did not bind to this protein [10,16]. We hypothesized that these medium-chain hydrocarbons could be components of the known trail pheromone analogs as well as side chains of piperidine alkaloids that possibly shift from full structures [37,38,39]. Perhaps, the alkane chains resemble by linking to the sixth position of piperidine alkaloids. Therefore, it is plausible that Sol g 2.1 might participate in either conveying an alkaloid component from its synthesis site to the venom reservoir or in creating complexes with the alkaloid within the venom duct [10,40,41]. In addition, regarding a previous report, the structural model of Sol i 2 showed that the C-terminal tail of the protein prevents access to the inner cavity compartments, resulting in solenopsin A not being able to access the protein interior. Nevertheless, from a computational prediction, they found that the elimination of the C-terminal tails enabled the venom alkaloid to bind effectively [16]. This evidence revealed that the C-terminal region plays a role in influencing the entry of ligands into the inner cavity of the PBPs related to conformation changes in an acidic environment [23,42].

In the in vitro binding assays with NPN, we found that the ligand with the highest affinity binding with rSol g 2.1 was decane, followed by undecane, dodecane, and tridecane. Interestingly, after applying the mixture of ligands, we found that the equilibrium dissociation constant, K_d_, of the mixture to Sol g 2.1 protein was prominently decreased when compared with individual ligands alone. This is due to a positive blend effect of the protein, wherein the mixture of ligands binds more strongly than the individual ligands. This finding is related to the equilibrium constant fitting with the Hill slope, which has a h value greater than 1.0, indicating that the protein and ligands have positive cooperativity [25].

The molecular docking of the endogenous alkanes to one internal and two external binding sites of Sol g 2.1 showed that the longer hydrocarbon chains, including dodecane and tridecane, had the highest affinity binding with the protein at both the internal and external binding sites. Nevertheless, from the competitive binding assay, decane had the strongest binding affinity with the rSol g 2.1 protein, followed by undecane, dodecane, and tridecane. Even though there were no significant differences in the K_d_ values of all ligands (Figure 3C), this is because the shorter hydrocarbon chains may easily move into the inner hydrophobic cavity of the protein, which may be blocked by the C-terminal region of the protein, preventing access to longer [43]. Related to the peak area ratio after approaching the binding assay, the result revealed that decane had the highest amount followed by undecane, dodecane, and tridecane, respectively, which is consistent with the affinities seen when in vitro. Interestingly, the computational model revealed that there are three binding sites on the Sol g 2.1. All of the ligands were most stabilized in the hydrophobic inner pocket of the protein by hydrophobic interactions. Therefore, a possible way to explain the positive blend effect of Sol g 2.1 with the mixture of endogenous hydrocarbons is that there is positive allostery between the external binding sites and the internal one. In the mixture, dodecane and tridecane may strongly bind at the external binding sites, whereas decane and undecane bind at the internal site [17,44]. Sol g 2.1 may bind to other cryptic ligands not detected here because it has some polar residues, including Ser and Tyr (Figure S4), which could interact with various functional groups; e.g., the residues equivalent to Ser58 and Ser46 of Sol i 2, Ser52, and Thr57 of LUSH (odorant-binding protein in Drosophila melanogaster) are in contact with the polar part of vaccenyl acetate via a hydrogen-bond donation from the amino acid OH to the pheromone [10,45].

Typically, the fire ant venom consists of piperidine alkaloids and pheromones. Both are insoluble forms that represent the most abundant components in venom (≥90%). Although some of the alkaloids bind to Sol g 2 proteins, which act as hydrophobic moiety protection, others possibly cannot bind [10,16]. Surprisingly, we found that the piperidine alkaloids we detected in the complete venom extract did not bind to Sol g 2.1 strongly enough to emerge bound to the protein from a gel-filtration column. We tested if the complete venom extract functions as a trail-marking pheromone and found that it does. Interestingly, the fully reconstituted venom (P+M+S), which was calculated between the protein and ligands while assuming a 1:1 ratio, had a higher ant-following response than crude venom. We believe that the mixture of hydrocarbons might bind with the protein, leading to a reduced evaporation rate of the hydrocarbon. Trail-following behavior is elicited by the alkaloids from crude venom [46,47], which is consistent with our results of the piperidine extraction, which resulted in trail-following responses. Importantly and unexpectedly, the reconstituted group of the mixture of hydrocarbons and Sol g 2.1 protein can also elicit the trail-following response. The composition between Sol g 2.1 and the mixture can entice the ants to follow the trails for a longer time and distance than the mixture in hexane only. Therefore, the Sol g 2.1 protein may act as a sticker, delaying the evaporation of the hydrocarbons.

## Figures and Tables

**Table 1:** Showing the elucidation of piperidine alkaloid compounds detected from *S. geminata* venom extraction by GC/MS.

Peak	Structure	Compound	RT (min) ^a^	Mass ^b^	Peak Area Ratio ^c^ (%)
1	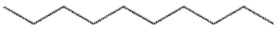	Decane	5.55	142	0.44
2	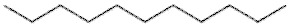	Undecane	7.86	156	0.21
3	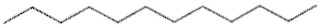	Dodecane	9.81	170	0.12
4	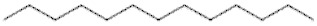	Tridecane	11.47	184	<0.1
5	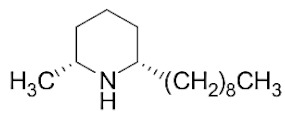	*cis*-2-methyl-6-nonylpiperidine	15.88	225.41	0.11
6	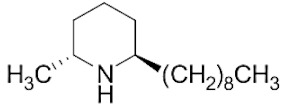	*trans*-2-methyl-6-nonylpiperidine	16.27	225.41	<0.1
7	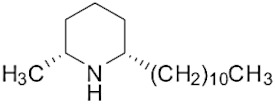	*cis*-2-methyl-6-undecylpiperidine	18.16	253.5	89.40
8	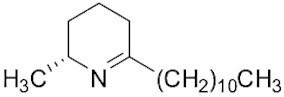	1,6-didehydro-2-methyl-6-undecylpiperidine	18.29	252	0.44
9	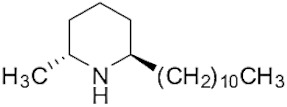	*trans*-2-methyl-6-undecylpiperidine	18.53	253.5	0.15
10	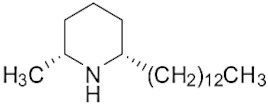	*cis*-2-methyl-6-tridecylpiperidine	19.03	281.5	9.20
11	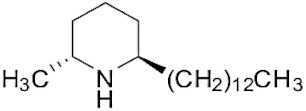	*trans*-2-methyl-6-tridecylpiperidine	19.89	281.5	0.10

^a^ Retention time (min) of peaks; ^b^ molecular mass of compounds (Da); ^c^ percent of peak area of compounds, which are shown in the GC chromatogram.

**Table 2:** Identification of MW of Sol g 2 protein contained in the flow-through after separating *S. geminata* crude venom by gel-filtration column.

Band	Matched Protein	Accession	−10 lgP ^a^	Average MW ^b^	Peptide Sequence	Coverage (%) ^c^	Species
C	Venom protein Sol g II	AAY32926.1	129.29	15,370	KDIAECARTLPK CENQPDDPLAR RGVFDDPAPAAIKKK	57	*S. geminata*

^a^ −10 lgP value was determined after LC-MS/MS analysis by PEAKS DB Software (PEAKS Studio 10.6); ^b^ an average MW of protein after the LC-MS/MS analysis; and ^c^ percent coverage of amino acid sequences.

## Data Availability

The data presented in this study are available from the corresponding author upon reasonable request.

## Supplementary Materials

The following supporting information can be downloaded at: https://www.mdpi.com/article/10.3390/molecules29051033/s1, Figure S1: Mass spectrums of peak 1–11 of the extraction of *S. geminata* crude venom using GC/MS analysis, Figure S2: SDS-PAGE with silver staining of the flow-through fractions of *S. geminata* crude venom after separation by gel filtration on P2 gel (size exclusion 100–1800 Da). M; protein marker, C; crude venom, T1; through-flow fraction 1 and T2; through-flow fraction 2, Figure S3: (A) Align the deduced amino acid sequence of Sol g 2.1 (GenBank: UYX46120.1), and Sol i 2 (*S. invicta*, PDB ID: 2ygu.1.A, 2.60 Å resolution). Asterisks (*) show cysteine conserved residue. (B) Superposition structure of Sol g 2.1 (turquoise;) and Sol i 2 (deep blue), Figure S4: Interactions between the amino acid residues of the top three protein binding sites and ligands by MOE. The ligands, including decane, undecane, dodecane, and tridecane were shown in (A–D), respectively. The green circles represent hydrophobic amino acids, the pink circles are the polar amino acids, and the circles, which are highlighted in blue are exposed to solvent, Video S1: Trail-Following Bioassay.
