# Supplementary material for: Protein–Ligand Binding and Structural Modelling Studies of Pheromone-Binding Protein-like Sol g 2.1 from Solenopsis geminata Fire Ant Venom
Source: Molecules. 2024 Feb 27;29(5):1033. doi: 10.3390/molecules29051033 (PMC10934024; doi:10.3390/molecules29051033)
Supplement: Supplementary file 1 [file molecules-29-01033-s001.zip › molecules-2833796-supplementary.pdf]

Supplementary Material

Protein–Ligand Binding and Structural Modelling Studies of Pheromone-Binding Protein-like Sol g 2.1 from *Solenopsis geminata* Fire Ant Venom

Siriporn Nonkhwao [1], Erika Plettner [2,*] and Sakda Daduang [1,3,*]

[1] Faculty of Pharmaceutical Sciences, Khon Kaen University, Khon Kaen 40002, Thailand; siriphorn_nonkhaow@kkumail.com

[2] Department of Chemistry, Simon Fraser University, Burnaby, BC V5A 1S6, Canada

[3] Protein and Proteomics Research Center for Commercial and Industrial Purposes (ProCCI), Khon Kaen University, Khon Kaen 40002, Thailand

* Correspondence: plettner@sfu.ca (E.P.); sakdad@kku.ac.th (S.D.); Tel.: +1-778-782-3586 (E.P.); +66-43-202-378 (S.D.)

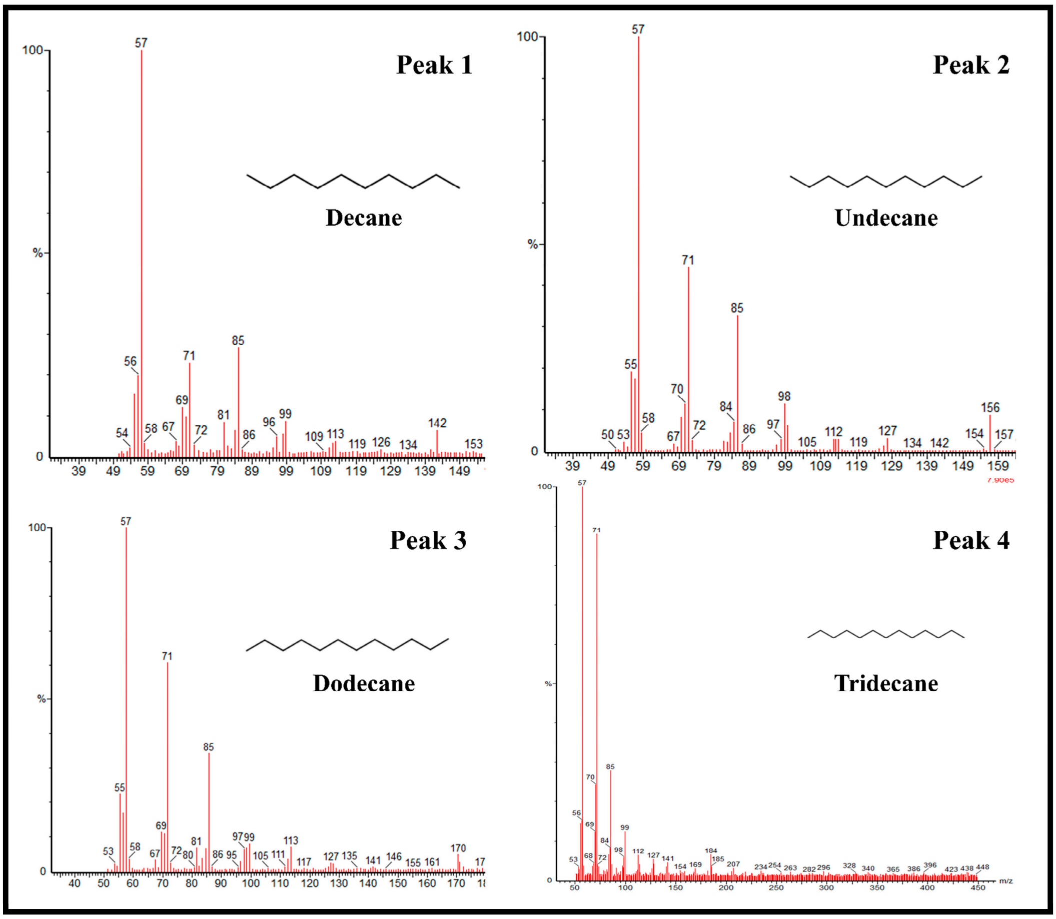

Figure S1. Cont.

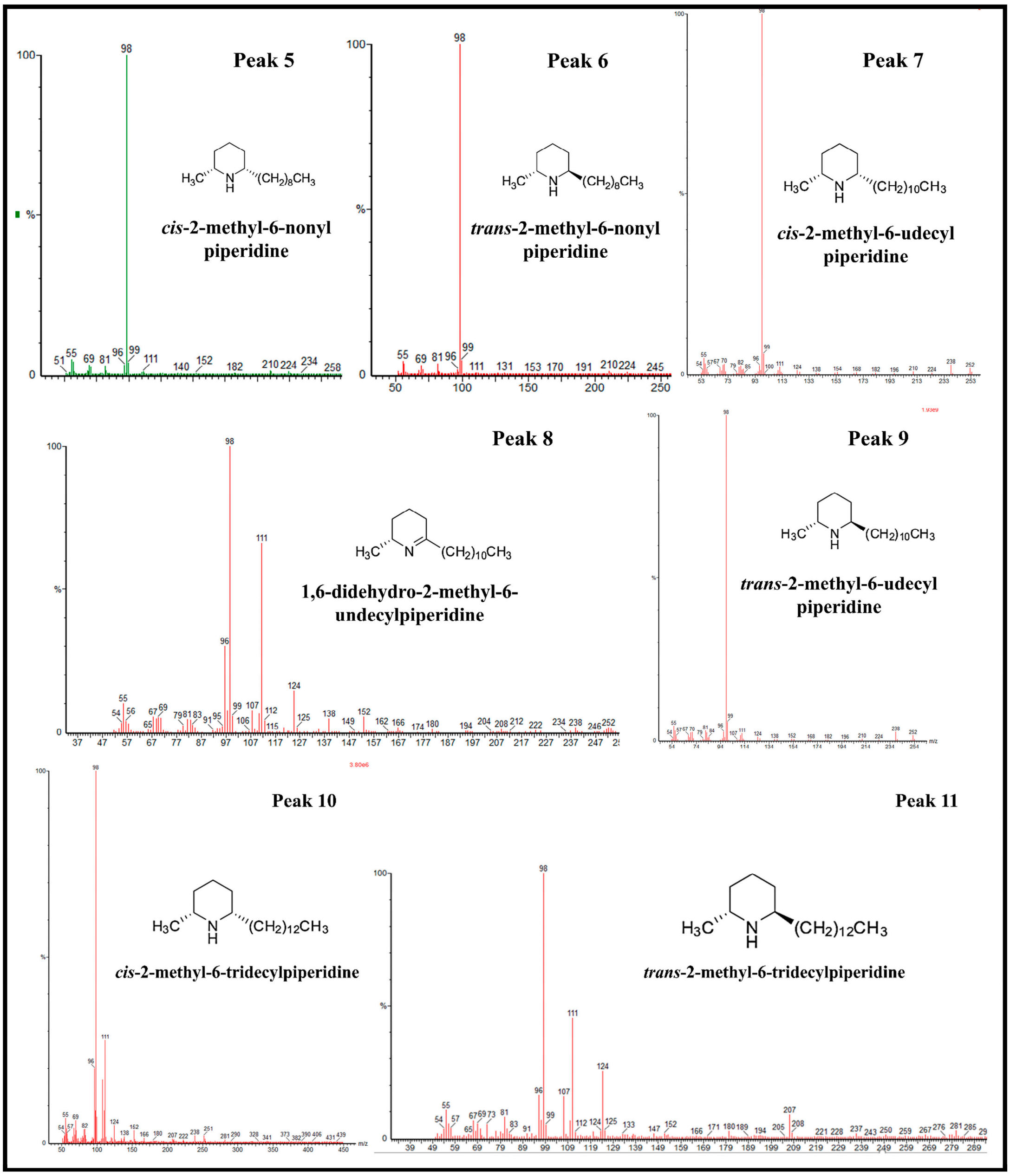

Figure S1. Mass spectrums of peak 1-11 of the extraction of *S. geminata* crude venom using GC/MS analysis.

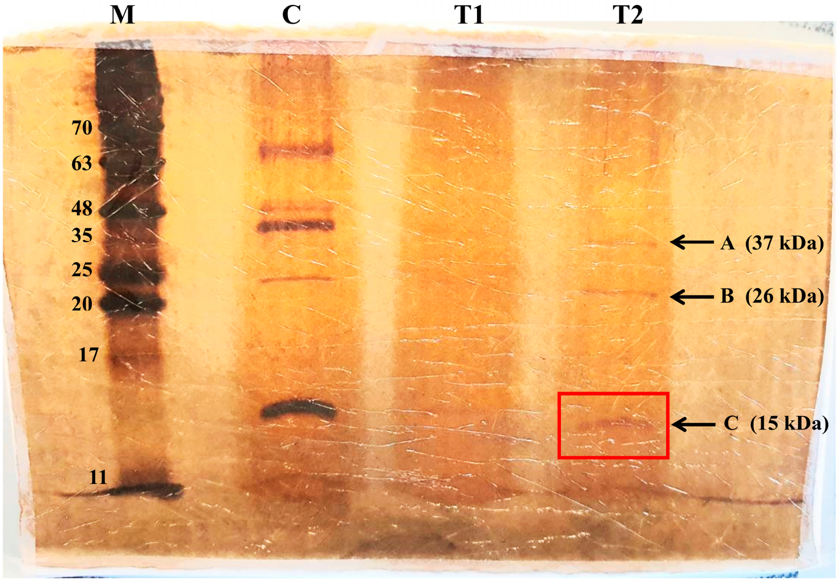

Figure S2. SDS-PAGE with silver staining of the flow-through fractions of *S. geminata* crude venom after separatio by gel filtration on P2 gel (size exclusion 100-1800 Da). M; protein marker, C; crude venom, T1; through-flow fraction 1 and T2; through-flow fraction 2.

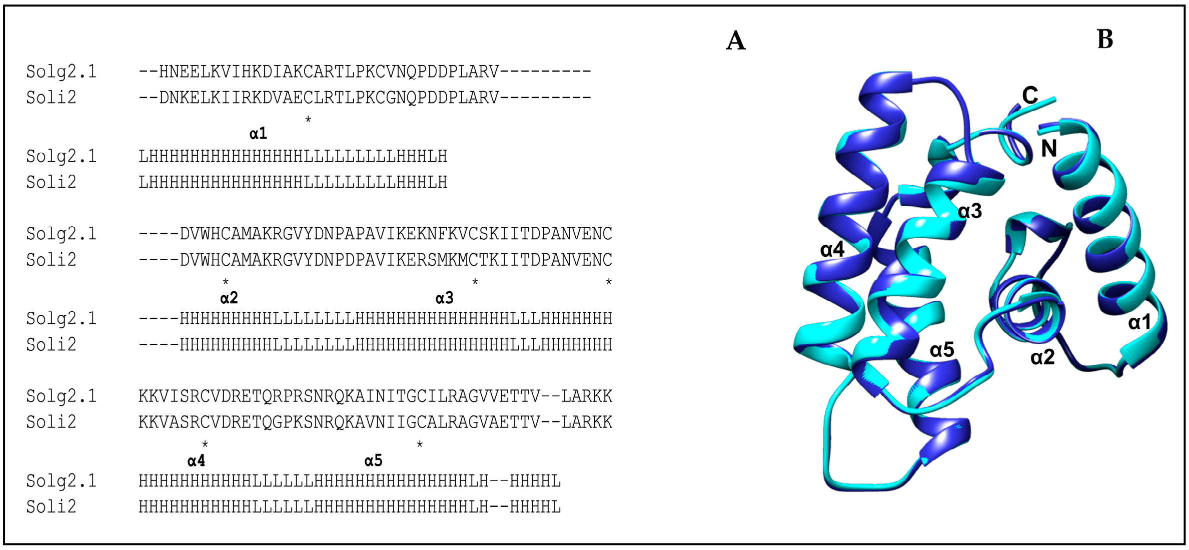

Figure S3. **(A)** Align the deduced amino acid sequence of Sol g 2.1 (GenBank: UYX46120.1), and Sol i 2 (*S. invicta*, PDB ID: 2ygu.1.A, 2.60 Å resolution). Asterisks (*) show cysteine conserved residue. **(B)** Superposition structure of Sol g 2.1 (turquoise;) and Sol i 2 (deep blue).

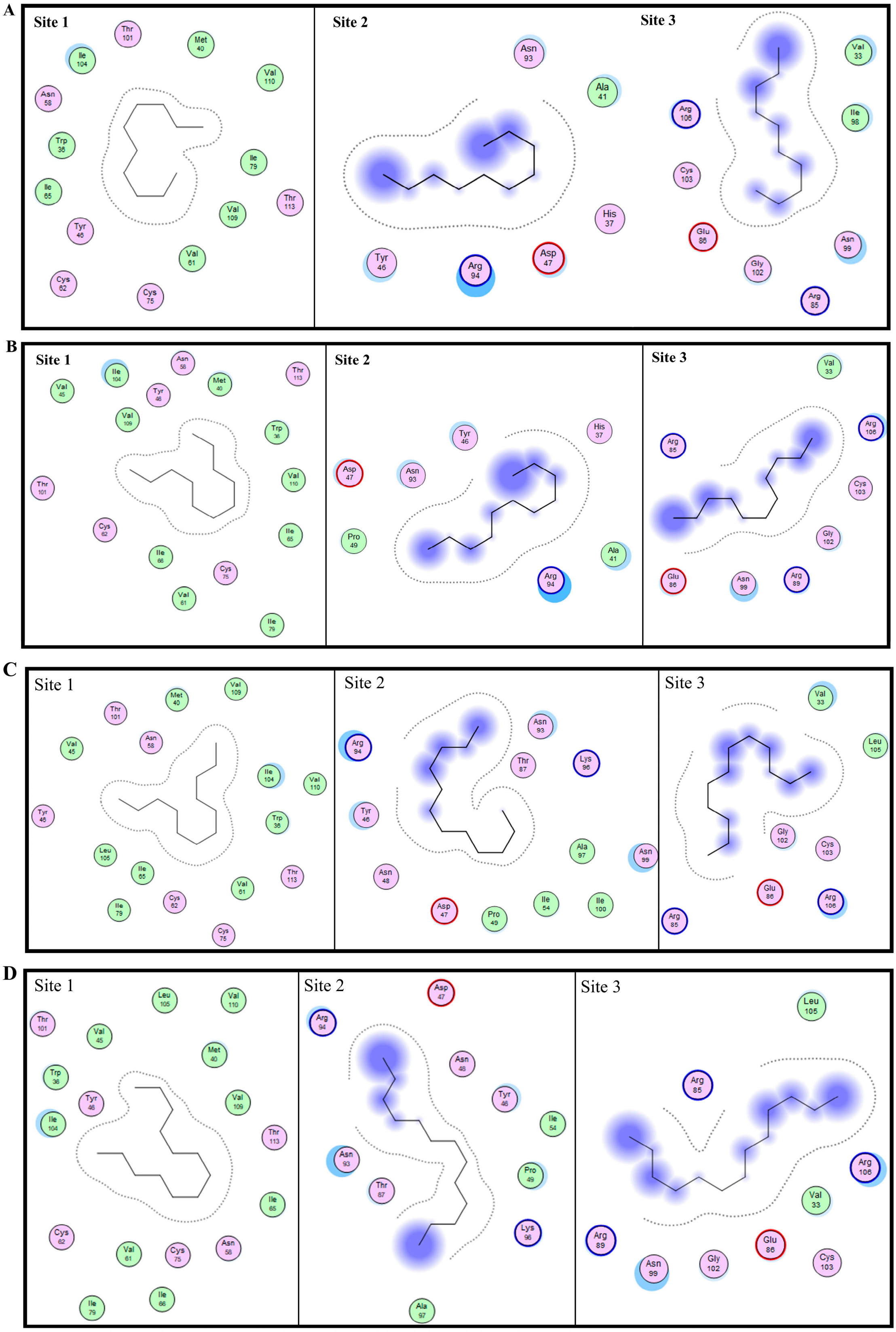

Figure S4. Interactions between the amino acid residues of the top three protein binding sites and ligands by MOE. The ligands, including decane, undecane, dodecane, and tridecane were shown in **(A–D),** respectively. The green circles represent hydrophobic amino acids, the pink circles are the polar amino acids, and the circles, which are highlighted in blue are exposed to solvent.
